# Effect of Retinohypothalamic Tract Dysfunction on Melatonin Level in Patients with Chronic Disorders of Consciousness

**DOI:** 10.3390/brainsci11050559

**Published:** 2021-04-28

**Authors:** Mikhail Kanarskii, Julia Nekrasova, Svetlana Vitkovskaya, Pranil Pradhan, Sergey Peshkov, Elena Borisova, Ilya Borisov, Olga Panasenkova, Marina V. Petrova, Igor Pryanikov

**Affiliations:** 1 Department for the Study of Chronic Disorder of Consciousness, Federal Research and Clinical Center of Intensive Care Medicine and Rehabilitology, 117647 Moscow, Russia; kanarmm@yandex.ru (M.K.); nekrasova84@yandex.ru (J.N.); svitkovskaya@fnkcrr.ru (S.V.); speshkov@fnkcrr.ru (S.P.); eborisova@fnkcrr.ru (E.B.); realzel@gmail.com (I.B.); opanasenkova@fnkcrr.ru (O.P.); mail@petrovamv.ru (M.V.P.); ipryanikov@fnkcrr.ru (I.P.); 2 Department of Anestesiology-Reanimatology, People’s Friendship University of Russia, 117198 Moscow, Russia

**Keywords:** melatonin, chronic disorder of consciousness, anoxia, traumatic brain injury, ophthalmology, retinohypothalamic tract

## Abstract

Objective: The aim of this study is to compare the secretion level of nocturnal melatonin and the characteristics of the peripheral part of the visual analyzer in patients with chronic disorders of consciousness (DOC). Materials and Methods: We studied the level of melatonin in 22 patients with chronic DOC and in 11 healthy volunteers. The fundus condition was assessed using the ophthalmoscopic method. Results: The average level of nocturnal melatonin in patients with DOC differed by 80% from the level of indole in healthy volunteers. This reveals a direct relationship between etiology, the level of consciousness, gaze fixation, coma recovery scale-revised score and the level of melatonin secretion. Examination by an ophthalmologist revealed a decrease in the macular reflex in a significant number of DOC patients, which in turn correlates negatively with the time from brain injury and positively with low values of nocturnal melatonin.

## 1. Introduction

Severe traumatic brain injuries (TBIs), cerebral vascular lesions (VLs), respiratory and cardiac arrest, and gross metabolic disorders often lead to a coma—a condition characterized by the absence of a qualitative and quantitative component of consciousness, structurally or functionally caused by damage to the ascending reticular formation or rostral midbrain or extensive lesions of the cerebral hemispheres. Coma is a transient state, its outcome can be either clear consciousness or, under less favorable circumstances, death or severe disorders of consciousness (DOC), such as vegetative state (VS) or minimally conscious state (MCS) [1,2]. In these conditions, the detection of minimal signs of consciousness or communication is of great importance. It thoroughly changes the clinical vector from palliative care to rehabilitation, makes it possible to personify cognitive rehabilitation, and gives relatives hope for a better outcome [3].

Currently, there is an active search for outcome predictors as well as for methods and techniques that contribute to the realization of the rehabilitation potential [4]. One of the markers of somatic preservation and consciousness dynamics, which has recently attracted much attention, are the features of circadian rhythms [5]. Despite the visualized preservation of the hypothalamus, which contains the main organism pacemakers in the suprachiasmatic nucleus (SCN), circadian rhythms in DOC patients differ significantly from the rhythms of healthy people [6]. Detection of melatonin is a reliable sign of circadian preservation [7]. The synthesis of melatonin is entirely dependent on the light-and-shadow background as well as on the integrity of the whole tract from the retina to the pineal gland. A special type of ganglion cell that expresses the light-sensitive pigment melanopsin represents the main peripheral component of this tract. These cells provide information on the intensity of light reaching the retina and direct their processes along the retinohypothalamic tract to the SCN of the hypothalamus [8]. Projections from the SCN to other nuclei of the hypothalamus, and further through endocrine influences and vegetative coverage of all tissues, provide an adaptive response of the body, depending on seasonal or daily fluctuations [9]. The descending projections from the hypothalamus along the posterior longitudinal bundle reach the preganglionic sympathetic neurons of the first thoracic segments of the spinal cord. Then, through the postganglionic afferent noradrenergic fibers in the *n. pinealis* along the vessels, they penetrate into the pineal gland and make contact with pinealocytes, synthesizing melatonin. The presence of melatonin receptors in almost all tissues explains the properties of melatonin as one of the key clock molecules of the body [10].

Currently, very few studies assess circadian rhythms in patients with chronic DOC. Among them, a number of papers are dedicated to the temperature curve [11], blood pressure, and heart rate [12]. The majority of studies are devoted to the sleep-wake cycle [5,13,14]; however, there are very few works that concern melatonin. In one of these studies, Belkin et al. [6] showed that the level of nocturnal melatonin in patients with DOC is reduced. The work of Guaraldi et al. [15] reveals the absence of light-induced suppression of melatonin. The authors explain the results as a possible dysfunction of the retinohypothalamic tract, but note that this issue requires further research. In [16], Oh et al. performed a prospective case-control study on individuals with pre-symptomatic Alzheimer’s disease (AD) pathology. Melanopsin-expressing retinal ganglion cell (mRGC) function was accessed using a standardized protocol of chromatic pupillometry. The authors suggest that mRGC dysfunction occurs in the pre-symptomatic AD stages, preceding cognitive decline.

The aim of this work was to assess the level of melatonin in patients with DOC of various etiologies in combination with an ophthalmological examination to assess the visual integrity of the retina.

## 2. Materials and Methods

### 2.1. Study Participants

The main group of study participants consisted of 22 patients with DOC (see Table 1). The control group consisted of 11 healthy volunteers from among the employees of the Federal Research and Clinical Center of Intensive Care Medicine and Rehabilitology of the Russian Federation.

The inclusion criteria assumed the presence of impaired consciousness to the extent of VS and MCS due to traumatic brain injury (TBI) or anoxic brain injury (ABI). The time from the event that caused the DOC was at least one month.

The exclusion criteria comprised brain stem damage, left hemispheric ischemic strokes, hemodynamic instability, mechanical ventilation at the time of the study, mental illness (for example, schizophrenia) in the patient’s medical history, and patient age of over 80 years. None of the patients was constantly receiving antipsychotics, antidepressants, β-blockers, α-blockers, nonsteroidal anti-inflammatory drugs, serotonin reuptake inhibitors, monoamine oxidase inhibitors or antiepileptic drugs. To exclude lesions to brainstem, hypothalamus, and visual tracts and to confirm the diagnosis of DOC, CT and/or MRI scans were performed.

A group of certified neurologists assessed the level of consciousness according to the Russian version of CRS-R Guidelines [17]. The results of the assessment are summarized in Table 1.

In healthy volunteers, we excluded neuro-ophthalmic pathologies as well as an intake of β-blockers, α-blockers, nonsteroidal anti-inflammatory drugs, serotonin reuptake inhibitors, antipsychotics, antiepileptic drugs, monoamine oxidase inhibitors, and melatonin-containing drugs. All volunteers worked on day shifts, had no jet lag in the previous 2 weeks, and had no complaints of sleep disturbances.

### 2.2. Data Collection

Blood samples were taken at 2:00 a.m. The light level in the ICU was monitored overnight using photoresistor GL5528 placed near the patient. The lowest light level at night (from 21:00 to 06:00) was from 0.15 to 1 lux. The highest level registered with lights on was near 100 lux; however, we did not allow episodes of turning on the light during the experiment. The specified light level was kept constant for all patients.

Prior to the blood sampling procedure, most of the patients had their eyes closed, except patients 1, 8, 14, and 17. However, due to the difficulty of reliably identifying sleep-wake cycles in patients with a low level of consciousness, closed eyes did not allow making an unambiguous conclusion as to whether these patients were asleep at the time of blood sampling. Healthy volunteers were exposed to similar conditions with the lights off. They were intentionally awakened to take biomaterials.

The measurement of melatonin content in blood serum was carried out using the method of tandem mass spectrometry in conjunction with ultra-performance liquid chromatography in the Clinic of New Medical Technologies “ArchiMed”, Moscow, Russia. The method included the determination of melatonin in the extract on an AB SCIEX QTRAP 5500 (AB SCIEX, Concord, ON, Canada) tandem mass spectrometer with triple quadrupole and ion trap, equipped with an atmospheric pressure chemical ionization (APCI) source controlled by Analyst software, version 1.6.2, AB Sciex Pte.Ltd, Framingham, USA. Nebulizer current was 2 mA, and source temperature was 450 °C. Melatonin was monitored in a positive mode using optimized parameters of ion transitions (MRM). Deuterium-labeled melatonin (melotonin-d6) was used as an internal standard. The measurement results were processed using the MultiQuant software Version 3.0.1, AB Sciex Pte.Ltd, Framingham, MA, USA.

We used direct ophthalmoscopy to assess the condition of the fundus, particularly the macular (ML) reflex. The qualified ophthalmologist that carried out the assessment considered ML reflex reduced when the color changed to yellow; the color uniformity and the reflectivity of the macular zone decreased compared to the group of healthy volunteers.

### 2.3. Data Processing

The accumulation, correction, and systematization of the initial information and visualization of the results obtained were carried out using STATISTICA 10 (StatSoft, Inc., Tulsa, OK, USA).

Nominal data were described with absolute values and percentages. To check the nature of the distribution of interval variables, the nonparametric Kolmogorov-Smirnov test was used. The analysis of the statistical significance of differences in quantitative traits for the two independent groups was performed using the Wilcoxon W-test. Differences were considered statistically significant at *p* < 0.05.

In order to study the relationship between the phenomena represented by the quantitative data, the distribution of which differed from normal, a nonparametric method was used—the calculation of the Spearman’s rank correlation coefficient. The values of the correlation coefficient were interpreted in accordance with the Chaddock scale for assessing the direction and strength of the correlation.

### 2.4. Legal Issues

Informed consent to clarify the details of the study was obtained in all cases. Since it was known beforehand that patients would not be able to express their consent to participate in the study, the document of consent was certified by three signatures from Ethics Committee members of the Federal Research and Clinical Center of Intensive Care Medicine and Rehabilitology. The Ethics Committee is an independent authority and makes decisions about the conduct of research in an impartial manner.

The study was carried out in accordance with the Declaration of Helsinki of the World Medical Association, approved at the 18th General Assembly of the WMA (Helsinki, Finland, June 1964), as amended by the 59th General Assembly of the WMA (Seoul, October 2008); Constitution of the Russian Federation, Art. 21; Fundamentals of the legislation of the Russian Federation on the protection of the health of citizens, orders and instructions of the Ministry of Health of the Russian Federation. The Ethics Committee approved the study on 23 December 2020, protocol no. 5/20/4.

The authors declare no conflict of interest.

## 3. Results

### 3.1. Demographic and Clinical Data

This section presents the demographic and clinical characteristics of patients with DOC who participated in the study (see Table 1).

We made equal samples of participants according to the etiology of the DOC, with 11 patients each, reflecting anoxic and traumatic brain injury.

**Figure 1:**
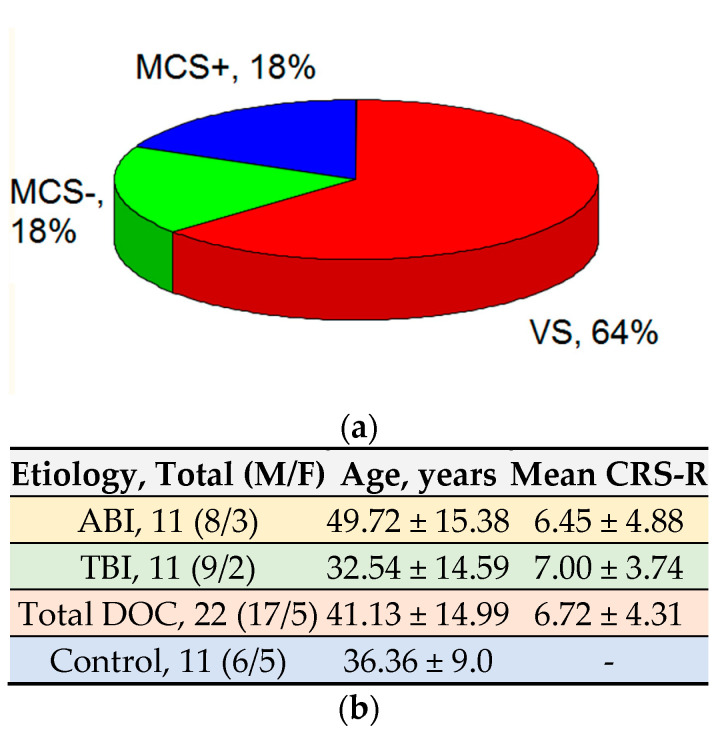
Classification of research participants (**a**) by the level of consciousness and (**b**) by etiology, age, sex, and CRS-R score.

For the level of consciousness, 14 patients were in the VS, four in MCS-, and four in MCS+ (Figure 1).

The average age of the patients at study entry was 41.13 ± 14.99 and the average score on the CRS-R scale was 6.72 ± 4.31 points.

The average age of the healthy volunteers was 36.36 ± 9.00, which generally corresponds to the age of patients in the main study group.

**Figure 2:**
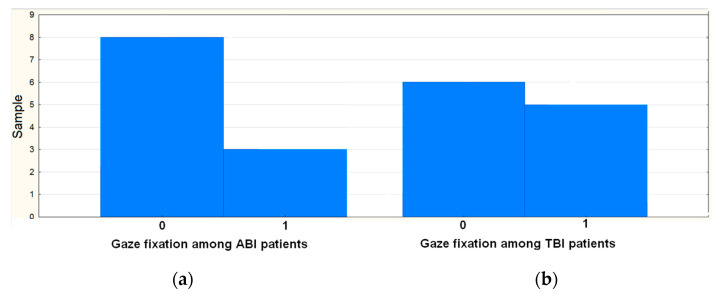
Fixation of gaze in patients (**a**) with TBI and (**b**) with ABI. Here, 0 means that a patient does not fixate the gaze and 1 means that a patient fixates the gaze.

Among the clinical indicators of the study participants, we paid special attention to the gaze fixation (Figure 2). Among patients with DOC due to TBI, 45.5% of participants fixed their gaze; among patients with consequences of global ischemia, 27.3% fixed their gaze.

The mean duration of the disease at the beginning of the study was 2.87 months, with a standard deviation of 2.41.

### 3.2. Melatonin Level

**Figure 3:**
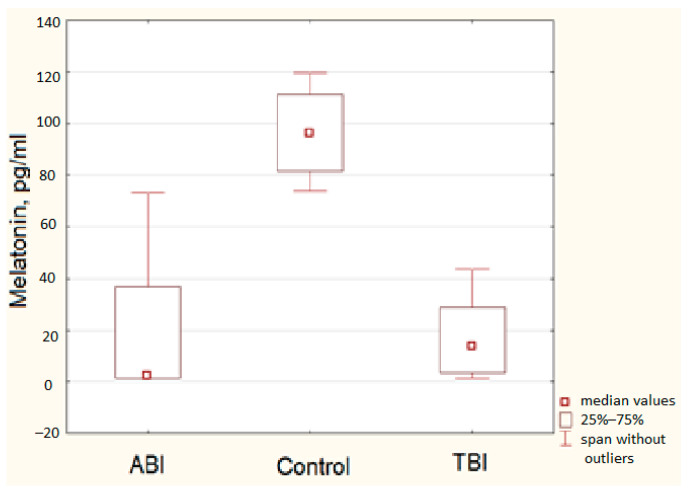
Range diagrams of melatonin levels depending on the etiology of the disease.

The average level of nocturnal melatonin in all participants with DOC was 18.54 ± 21.20 pg/mL, while in healthy volunteers it was 97.58 ± 13.07 pg/mL. At the same time, the median value for patients with ABI was 2.4 pg/mL and for patients with TBI was 13.80 pg/mL, which allows us to conclude that there is a direct strong relationship between the etiology of the disease and the level of melatonin secretion (Figure 3). Spearman’s rank correlation coefficient for these variables is *r_s_* = 0.73 at *p* < 0.05.

A direct strong relationship was found between the amount of melatonin and the level of consciousness (*r_s_* = 0.86 at *p* < 0.05). There was a direct moderate correlation between the level of melatonin and CRS-R score (*r_s_* = 0.65 at *p* < 0.05) as well as between the level of melatonin and the patient’s ability to fixate the gaze (*r_s_* = 0.70 at *p* < 0.05). However, it must be noted that level of consciousness and CRS-R score were in turn strongly related, with *r_s_* = 0.87 at *p* < 0.05. The above correlation essentially reflects the relationship between the level of consciousness and the scale by which it is assessed. Moreover, the CRS-R scale is just a numerical reflection of the level of consciousness. The finding does not contradict the general trend regarding the relationship between the level of melatonin and consciousness.

At the same time, a strong negative correlation was found between the amount of melatonin and the time from the event that caused the DOC to the study (*r_s_* = −0.84 at *p* < 0.05). We found no association between the age of the participants and the amount of melatonin in this study.

Ophthalmological examination revealed a decrease in the ML reflex in ABI patients in 90.91% of cases. In patients with TBI, a decrease in the ML reflex was observed in 36.36% of cases. We revealed no disruption of the ML reflex in the control group of healthy volunteers. The incidence of ML reflex disorders also moderately negatively correlated with the time from brain damage to examination; thus, the likelihood of reflex disturbances increases with time.

A strong direct relationship (*r_s_* = 0.75 at *p* < 0.05) was found between gaze fixation and the ML reflex: patients with intact retinal function fixate their gaze more often than patients with a reduced reflex. Associated with this fact is the observed direct moderate correlation between the CRS-R score and the ML reflex, with *r_s_* = 0.62 at *p* < 0.05.

Finally, the level of serum nocturnal melatonin directly correlates with the degree of the ML reflex disruption: *r_s_* = 0.65 at *p* < 0.05.

## 4. Discussion

The present study confirmed a decreased melatonin secretion at night in patients with DOC compared with healthy volunteers [7,15]. This is possibly due to dysfunction of the retinohypothalamic tract at any of its sites. However, it should be noted that, according to MRI data, the structures of the hypothalamus in the patients of the main group were not damaged. In this study we can see that in patients at an early stage of central nervous system damage, the function of the retinohypothalamic tract is generally preserved, which is confirmed by the results of an ophthalmological study and relatively high rates of melatonin secretion in the early period of the disease.

In patients with a higher level of consciousness, the secretion of melatonin is maintained in a range close to normal, which makes melatonin level an indirect indicator of the severity of the lesion. This statement is true both for patients with anoxic etiology and for patients with TBI.

In patients with anoxic etiology and, accordingly, homogeneous damage to the cerebral cortex, we observe a lower secretion of melatonin, which can be explained by a transdegenerative process (atrophy due to inactivity) between the visual cortex, thalamus, and retina. Among patients with TBIs, there is also a correlation between the severity of the brain injury and the level of melatonin, as evidenced by the results of neuroimaging (CT and/or MRI scans of the brain).

The statement that low melatonin is associated with usual bright lighting of the intensive care units at nights is dubious in light of the obtained data.

It is important to emphasize that gaze fixation in patients with low levels of consciousness is probably limited by the factors listed above. We assume that, in part, the discrepancies in the results of studies on gaze fixation may be mediated by degenerative processes along the entire path of the visual analyzer [18,19,20,21]. Thus, gaze fixation without additional ophthalmic and, possibly, laboratory studies should be used with caution to differentiate between VS and MCS.

It can be concluded with caution that the use of melatonin in combination with other instrumental diagnostic methods can contribute to a more accurate divergence between VS and minimal consciousness, which is undoubtedly extremely important in determining rehabilitation potential.

A number of limitations are inherent in this study. First is the lack of high-precision instrumental diagnostic methods during ophthalmological examination, the application of which to bedridden patients in our case was difficult. It is also necessary to note an insufficiently large sample of patients, etiology restrictions on TBI and ABI patients only, and a single biomaterial sampling, which did not allow a full assessment of the secretory capacity of the pineal gland throughout the observed period. The small sample of patients is also an issue; however, since DOC is a rather rare condition, a sample of 22 people, in our opinion, is acceptable. Another limitation is also the single sampling of biomaterial, which does not allow detecting circadian changes in the level of melatonin secretion. The authors consider this a future task.

## 5. Conclusions

The aim of this work was to assess the effect of dysfunction of the retinohypothalamic tract on the level of melatonin in patients with chronic DOC. According to the results of the study, we revealed a correlation between the low secretion level of melatonin and the severity of brain injury with possible damage to the retina through the transdegenerative mechanism.

More comprehensive quantitative and instrumental studies are required to obtain more reliable results. Thus, research is needed to address the problem of gaze fixation in DOC patients, to estimate the value of melatonin for differential diagnosis of the level of consciousness, and to reveal the process of atrophy due to inactivity.

## Figures and Tables

**Table 1:** Demographic and clinical indicators of study participants.

No.	Age	CRS-R	Time from Brain Injury to Study Entry, Month	Etiology	Level of Consciousness	Melatonin Level, pg\mL	Gaze Fixation	ML—Reflex
1	72	5	1	ABI	VS	73.2	0	Reduced
2	60	4	9	ABI	VS	<1.3	0	Reduced
3	32	5	7	ABI	VS	<1.3	0	Reduced
4	65	3	2	ABI	VS	5.1	0	Reduced
5	45	4	8	ABI	VS	5.5	0	Reduced
6	75	3	7	ABI	VS	<1.3	0	Reduced
7	39	3	7	ABI	VS	<1.3	0	Reduced
8	37	17	2	ABI	MCS+	68.4	1	Reduced
9	44	7	3	ABI	MCS-	<1.3	1	Reduced
10	36	5	3	ABI	VS	36.7	0	Reduced
11	42	15	5	ABI	MCS+	30.5	1	Normal
12	30	5	2	TBI	VS	4.8	0	Normal
13	26	4	5	TBI	VS	13.8	0	Reduced
14	18	8	8	TBI	MCS+	35.3	1	Normal
15	58	4	4	TBI	VS	2.3	0	Reduced
16	18	5	2	TBI	VS	3.4	0	Normal
17	21	7	3	TBI	MCS-	22.7	1	Normal
18	55	5	3	TBI	VS	6.7	0	Reduced
19	46	4	6	TBI	VS	1.2	0	Reduced
20	37	8	2	TBI	MCS-	19.7	1	Normal
21	20	7	5	TBI	MCS-	28.7	1	Normal
22	29	13	1	TBI	MCS+	43.6	1	Normal
23	41	-	-	Control	Conscious	92.0	1	Normal
24	39	-	-	Control	Conscious	73.9	1	Normal
25	29	-	-	Control	Conscious	118.9	1	Normal
26	24	-	-	Control	Conscious	81.5	1	Normal
27	41	-	-	Control	Conscious	105.8	1	Normal
28	41	-	-	Control	Conscious	88.4	1	Normal
29	22	-	-	Control	Conscious	104.4	1	Normal
30	40	-	-	Control	Conscious	81.5	1	Normal

## Data Availability

The data are not publicly available due to the nature of this research because all the data for this paper is available in the article itself in the form of a table.
